# Evaluation Model of Autonomous Vehicles’ Speed Suitability Based on Overtaking Frequency

**DOI:** 10.3390/s21020371

**Published:** 2021-01-07

**Authors:** Shiwu Li, Mengyuan Huang, Mengzhu Guo, Miao Yu

**Affiliations:** 1 School of Transportation, Jilin University, 5988 Renmin Street, Changchun 130022, China; shiwu@jlu.edu.cn (S.L.); huangmy19@mails.jlu.edu.cn (M.H.); yumiao17@mails.jlu.edu.cn (M.Y.); 2 State Key Laboratory of Automotive Simulation and Control, Jilin University, 5988 Renmin Street, Changchun 130022, China

**Keywords:** overtaking frequency, autonomous vehicles, hierarchical judgment, vehicle speed suitability, millimeter-wave radar, intelligent driving system

## Abstract

Speed judgment is a vital component of autonomous driving perception systems. Automobile drivers were able to evaluate their speed as a result of their driving experience. However, driverless automobiles cannot autonomously evaluate their speed suitability through external environmental factors such as the surrounding conditions and traffic flows. This study introduced the parameter of overtaking frequency (OTF) based on the state of the traffic flow on both sides of the lane to reflect the difference between the speed of a driverless automobile and its surrounding traffic to solve the above problem. In addition, a speed evaluation algorithm was proposed based on the long short-term memory (LSTM) model. To train the LSTM model, we extracted OTF as the first observation variable, and the characteristic parameters of the vehicle’s longitudinal motion and the comparison parameters with the leading vehicle were used as the second observation variables. The algorithm judged the velocity using a hierarchical method. We conducted a road test by using real vehicles and the algorithms verified the data, which showed the accuracy rate of the model is 93%. As a result, OTF is introduced as one of the observed variables that can support the accuracy of the algorithm used to judge speed.

## 1. Introduction

In the past few decades, large amounts of research have been undertaken on the development of driverless automobiles [1]. However, the challenge of the perception and evaluation of autonomous decision-making in driverless automobiles is still difficult to resolve. If the final decision and the process of action control can be implemented steadily and reliably, an automobile will avoid traffic accidents and therefore reduce traffic. Even though a number of ideas regarding the dynamic programming of an algorithm for the decision-making process of human-driven vehicles have been expressed, the capacity to perceive the driving status of a driverless automobile before the final decision-making process is still a challenge for researchers.

Traffic safety technical experts have proposed many methods for the evaluation of automobile safety. Based on the deterministic vehicle accident model, some researchers have investigated the safety of a vehicle while taking into account the uncertainty of key variables, including vehicle parameters, the vehicle velocity, wind velocity, road friction coefficient, and superelevation [2,3,4,5]. Based on this research, Hou et al. [6] added the highway system and other environmental conditions into their research and proposed a comprehensive evaluation framework to evaluate the safety performance of single-vehicle traffic in a random traffic flow under dangerous driving conditions. Laugier et al. [7] analyzed typical urban driving dynamic scenes when evaluating vehicle safety. However, the disadvantage of the above research is the single data collection method, and the state of the vehicle is only evaluated at the single car level. At the same time, the approach does not consider the situation of smart vehicles compared with vehicles in the other lanes. It is not easy to achieve this evaluation at the multi-vehicle level.

Experts have researched driver decision-making for decades and have made great progress. Some researchers have studied the factors that influence driver decision-making [8]. The general factors include the traffic environment and the driver’s physiology and psychology, driving skills, perceptual reaction time, etc. Some researchers have developed models of driver decision-making behavior based on the above influencing factors [9,10]. Based on previous studies, Dovgan et al. [11] combined the driver model with the goal optimization of travel time cost and fuel consumption and proposed a human-like intelligent driving strategy. Gu established a human-like path-planning model to describe the driver’s evaluation of the road environment and the control of the vehicle in an intersection scene with a signal [12].

Global domain experts have also researched the factors that need to be considered for driverless automobiles to make decisions. Duan proposed a hierarchical reinforcement learning method for self-driving vehicle decisions, which was applied to a highway driving scene [13]. The Carnegie Mellon Navlab vehicles combine a rationally distributed system (PolySAPIENT) with a novel evolutionary optimization strategy (PBIL) and use route-level planning to achieve context-sensitive local decision-making and complex motion planning, using reasonable behavior theory to decide driving behavior [14,15,16,17]. Based on the perceived traffic characteristics, the decision-making system for autonomous vehicles researched by Stanford University converts one driving status to another [18,19,20]. Li et al. proposed a human-like decision-making system based on on-board sensors and the use of a convolutional neural network model to detect, identify and extract road environment information [21]. Zhu et al. proposed a human-like autonomous car following a planning framework based on deep reinforcement learning and established a human-like car-following model [22]. In addition, some driving decisions based on learning algorithms have been extensively studied in recent researches [23,24].

Based on subjective factors such as traditional vehicle drivers’ perceptions of a vehicle’s condition, humans can make corresponding judgments on whether the vehicle’s driving status is safe. However, for a driverless automobile, the limitation of external information (especially the limitation of long-distance information) will affect the in-vehicle ECU (Electronic Control Unit)’s judgment on the vehicle’s speed suitability. Quantifying the driving state of intelligent vehicles in the traffic flow requires a specific judgment indicator. Thus, it is necessary to introduce certain parameters to evaluate the driving state of intelligent vehicles.

In this paper, we proposed a hierarchical algorithm to assess the rationality of the driving speed of auxiliary self-driving vehicles, which can be used to better improve the autonomous driving perception system. 

The main contribution is summarized as follows:

- We evaluate the discrepancy of driving states between autonomous vehicles and other vehicles in the traffic flow; to this end, the overtaking frequency (OTF) is proposed.
- Based on OTF and vehicle operating parameters, we propose a hierarchical algorithm that integrates horizontal and vertical safety assessments to inherit the advantages of both multi-lane and single-lane information.
- By incorporating GPS into the Controller Area Network (CAN) bus of a millimeter-wave radar, software for detecting vehicles that are overtaking by/overtaken by on both sides of an autonomous vehicle is developed, which allows the recording of OTF.

The remainder of the paper is organized as follows. Section 2 proposes the OTF parameters and details the definitions and acquisition methods of OTFs, as well as a model for evaluating speed suitability based on OTF hierarchy. Section 3 provides a complete explanation of the experimental procedure, as well as the equipment used and the parameters acquired. The performance of the proposed method is verified in Section 4. Finally, the paper is summarized in Section 5.

## 2. Methods

### 2.1. OTF

#### 2.1.1. Establishment of the OTF Factor

Driving behaviors such as rapid acceleration, rapid deceleration, lane changes and overtaking are generally preceded by an incentive that triggers the driver to change their road position, such as cutting in front of a vehicle or braking in front of a vehicle. In this case, the driver determines if the traffic in the lane is consistent with the traffic in the neighboring lanes and ultimately decides whether to change the driving status of the vehicle. Thus, the safety of the surrounding environment has an important impact on whether the driver intends to change the behavior of the vehicle. In this paper, OTF is defined to evaluate the flow state of vehicles in the side lane compared to the flow state of the main vehicle.

The definition of “overtaking frequency” is the difference between the number of vehicles overtaking and the number of vehicles being overtaken in the left and right lanes of a driving vehicle at a fixed unit time interval.

Since the OTF is a parameter that reflects the difference in speed between the vehicles in both lanes and the host car, it is related to the difference in the number of vehicles overtaking and being overtaken on both sides. We set Nr as the difference in the number of vehicles overtaking and being overtaken on both sides. This article only discusses the three-lane scenario, and the formula to define Nr is shown in Equation (1):

(1) $$N_{a}-N_{b}=Nr$$

where $N_{a}$ represents the number of vehicles in the left and right lanes passing the host car, and $N_{b}$ represents the number of vehicles in the left and right lanes passed by the host car.

We establish OTF as a formula for speed, time and the difference between the number of vehicles passing and being passed on either side, as shown in Equations (2) and (3):

(2) $$\mathrm{OTF}=f_{1}\left(Nr,v,\delta \right)$$

where δ represents the time window, v represents the velocity of the host vehicle and Nr represents the difference in the number of vehicles passing and being passed on both sides.

Given the definition of OTF, the formula for calculating OTF is shown in Equation (3)

(3) $$\mathrm{OTF}=\frac{N_{a}-N_{b}}{\delta }=\frac{Nr}{\delta }$$

The value of the time window varies for different velocities. As the velocity increases, it is necessary to take a longer time window to obtain the OTF in order to obtain a more accurate assessment, as shown in Equation (4).

(4) $$\delta \{\begin{array}{c} v_{1}\\ v_{2}\\ v_{3}\\ .\\ .\\ .\\ v_{n}\{\begin{array}{c} N1\\ N2\\ N3\\ .\\ .\\ .\\ Nr \end{array} \end{array}$$

#### 2.1.2. Acquisition of OTF

In this paper, we use millimeter-wave radar to detect whether there are other vehicles passing on both sides of the vehicle. The vehicle speed input from GPS can filter the stationary objects on the road to recognize whether the vehicle body has passed other vehicles on both sides.

1. Lateral vehicle recognition algorithm

The vehicle body coordinate system is established. The *x*-axis direction is the longitudinal direction of the moving vehicle, and the *y*-axis direction is the lateral direction of the moving vehicle.

The longitudinal relative distance $D_{x}$, lateral relative distance $D_{y}$, longitudinal relative velocity $v_{x}$, lateral relative velocity $v_{y}$ and ID of the target object relative to the host car are obtained from the millimeter-wave radar.

When $D_{y}>\;0$ is detected, the target is considered to be on the left side of the driving vehicle, and when $D_{y}<\;0$ is detected, the target is considered to be on the right side of the driving vehicle.

2. Lateral vehicle overtaking recognition

**Figure 1:**
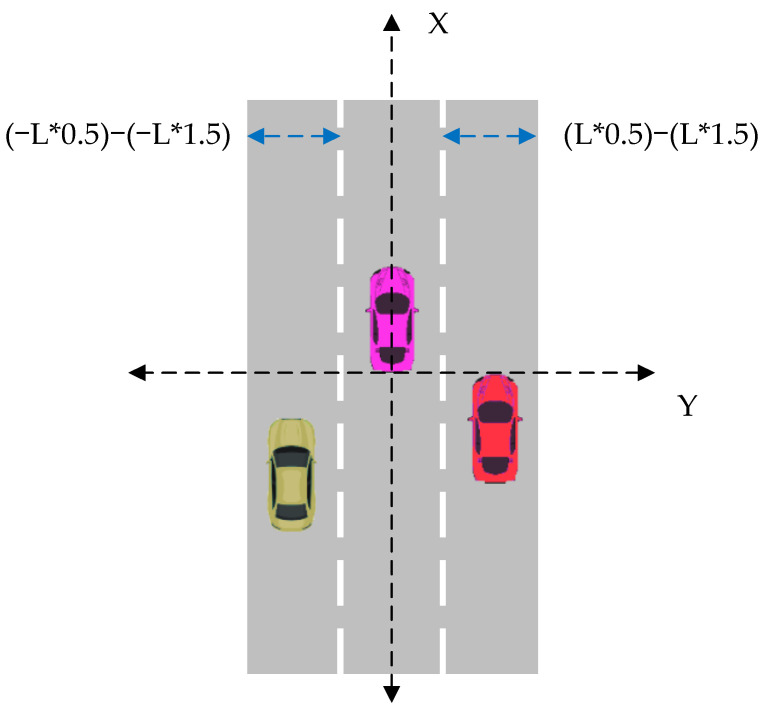
Schematic diagram of the algorithm for overtaking recognition in lanes on both sides of the main vehicle.

Since we want to detect the vehicle dynamics of the left and right adjacent lanes of the host car, we need to set a certain range. Firstly, we set the width of each road as L. When the target is detected in the left lane, $D_{y}$ ≤ (L+L/2) and the target $v_{x}$ ≥ 0, this is an indication of overtaking behavior from a vehicle in the left lane; when a target object is detected in the right lane, $D_{y}$ ≥ (L+L/2) and the target object $v_{x}$ ≥ 0, this is an indication of overtaking behavior by a vehicle in the right lane. The algorithm’s schematic diagram is shown in Figure 1.

3. Vehicle recognition algorithm for the host vehicle overtaking vehicles on the left and right sides

The difference between the longitudinal speed v of the main vehicle and the relative longitudinal velocity $v_{x}$ of the target relative to the main vehicle is the absolute velocity $v_{n}$ of the target, as detected by the millimeter-wave radar.

(5) $$v_{n}=v_{x}+v$$

In this paper, we set $v_{a}$ as the speed threshold. If the target speed is less than the threshold, the target is determined to be a non-vehicle target; when the speed is greater than the threshold and when the target is detected in the left lane and the lateral distance to the millimeter-wave radar $D_{y}$ ≤ (L+L/2), relative to the longitudinal velocity of the millimeter-wave radar, $v_{x}$ < 0, it is determined that the driving vehicle overtakes a target vehicle in the left lane. When a target is detected in the right lane and its lateral distance to the millimeter-wave radar is $D_{y}$ ≥ (L+L/2), and when the longitudinal velocity of the relative millimeter-wave radar $v_{x}$ < 0, it is determined that the driving vehicle overtakes a target vehicle in the right lane.

**Figure 2:**
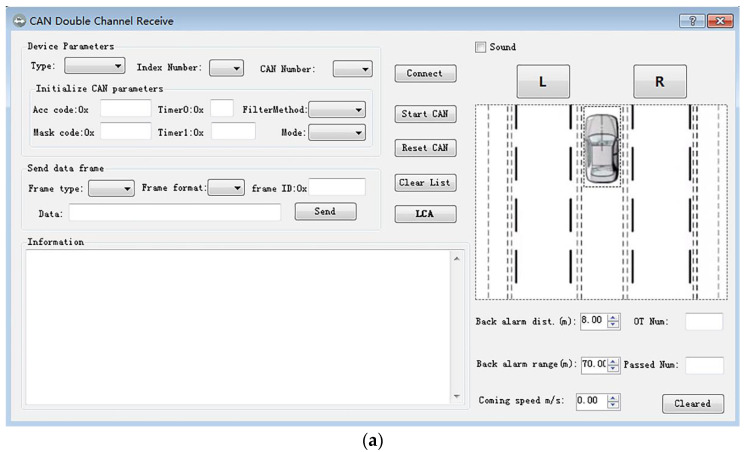

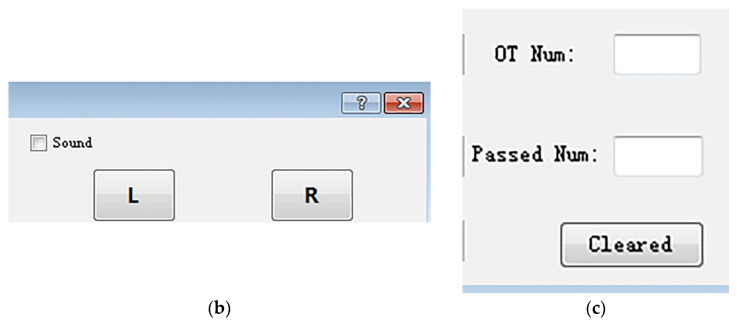
The detection software interface developed in this paper: (**a**) Software interface diagram, (**b**) Identify the keys to the left and right of the target, (**c**) Record the number of overtaking/overtaken vehicles.

Since this paper investigates speed suitability assessment in a highway scenario, the threshold $v_{a}$ is set to 60 km/h. The detection software developed based on the above algorithm is shown in Figure 2.

When a vehicle is detected on the left side, the “L” button will turn red. When a vehicle is detected on the right side, the “R” button will turn red. At the same time, the software also has the function of counting overtaking vehicles and the number of vehicles passed.

### 2.2. Speed Adaptation Evaluation Model for Different Situations

**Figure 3:**
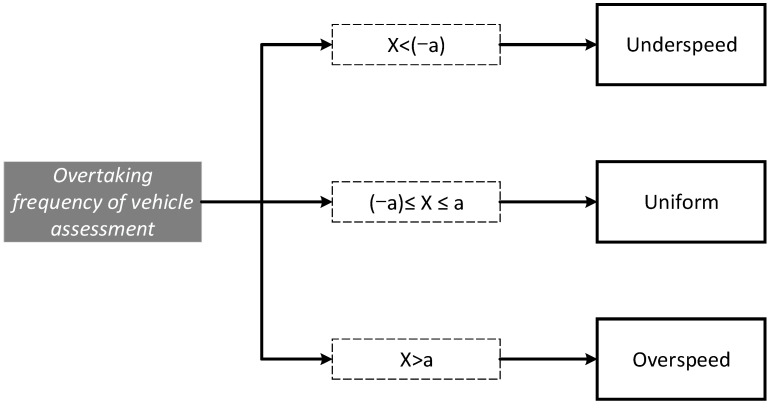
Judgment framework for overtaking frequency (OTF).

According to the Equation (3), when OTF = 0, the speed of the host car and the traffic flow of the two adjacent lanes are the same, that is, the difference is the smallest, but setting the threshold to 0 is too ideal. Thus, we set the threshold [−a, a]. When evaluated with OTF only, while the OTF belongs to [−a, a], the vehicle driving suitability is classified as appropriate. The judgment framework is shown in Figure 3. However, in the establishment of a method for evaluating vehicle suitability, it is not enough to simply use a threshold to divide speed suitability. Different scenarios should be discussed and an evaluation model should be established.

#### 2.2.1. Case 1: No Traffic in Front of the Host Vehicle

When the host car is driving in the current lane, since there is no vehicle in front of it, the autopilot vehicle needs to determine whether its driving status is appropriate and whether its speed is in line with the other vehicles in the flow of traffic, with the assistance of the vehicles in the left and right lanes. This is used to make the next adjustment to the driving state.

1. OTF ∈[−a, a]

In the free flow (no vehicle before or after), the speed selection of the vehicle in this lane is related to the speed limit conditions, road conditions, weather conditions, etc. This article does not consider the impact of weather, roads, etc. When OTF ∈[−a,a], the speed of the vehicle is compatible with the traffic speed in the left and right lanes, which means that the driving state of the vehicle is consistent with the driving state of the traffic in the neighboring lanes, and so the vehicle does not need to make much adjustment.

2. OTF <(−a) or OTF >(a)

In a complex traffic flow, the OTF parameter is created when the speed of the vehicle differs from the speed of the two adjacent lanes of traffic. When the OTF <(−a), the vehicles in the adjacent two lanes are overtaking the vehicle within a certain time window and the speed of the vehicle is too slow relative to the speed of the traffic in the adjacent two lanes, and therefore the speed of the vehicle should be adjusted to some extent in the absence of a vehicle in front. Similarly, when the OTF >(a), the speed should be reduced to an OTF close to the threshold.

#### 2.2.2. Case 2: Current Lane with Vehicles in Front

In addition to the driving state of the vehicle in front, the vehicle will make the final decision to accelerate, maintain speed, or slow down in conjunction with the movements of other vehicles in the adjacent lanes.

Due to the presence of a vehicle in front of the main vehicle in the current lane, the longitudinal driving risk and driving suitability of the main vehicle are closely related to the driving state of the front vehicle. It is well known that longitudinal driving risk is related to vehicle speed and longitudinal acceleration. In addition to that, two physical parameters can be considered as variables that affect the perception of longitudinal driving risk: one is the time headway (THW), which is obtained by dividing the headway distance by the speed of the main vehicle; the other is the time to collision (TTC), which is obtained by dividing the distance from the main vehicle by the relative speed between the two vehicles. THW and TTC are defined by Equations (6) and (7).

(6) $$\mathrm{THW}=\frac{D}{v}$$

(7) $$\mathrm{TTC}=\frac{v_{rel}}{D}$$

where D represents the headway distance, v represents the host vehicle’s velocity and $v_{rel}$ represents the longitudinal relative speed between the front car and the host car.

In this paper, four parameters, namely speed, THW, TTC, and $a_{x}$ (longitudinal acceleration of the host vehicle), are chosen to evaluate the reasonableness of the vehicle speed in a single lane.

1. OTF ∈[−a, a]

When the OTF of the main vehicle is ∈[−a, a], the speed of the vehicle corresponds to the speed of the traffic in the two adjacent lanes. In the case in which the speed of the vehicle is consistent with the overall traffic flow of the three lanes, the speed selection and safety state of the vehicle is related to the driving state of the preceding vehicle. Therefore, the reasonableness of the speed at this time should be evaluated using the single-lane longitudinal risk evaluation method.

2. OTF <(−a) or OTF >(a)

When the speed of the host vehicle is different from the speed of the vehicles in the two adjacent lanes, the vehicle evaluation scheme is different in the case in which there is no vehicle in front of the host vehicle, because there is a vehicle ahead.

When the OTF <(−a), it means that the current speed of the vehicle is too slow relative to the other lanes of traffic, which is also an unsafe condition. Therefore, the four parameters described in Equation (1) should also be considered to determine whether the current speed is appropriate and safe for the vehicle. However, if the current driving condition is evaluated to be inappropriate due to an obstruction by the vehicle in front, the vehicle should choose to change lane measure.

When the OTF >(a), the vehicle should reduce its speed according to the OTF evaluation to an OTR close to the critical value when the speed is evaluated as reasonable in the single-lane longitudinal safety evaluation. In the case of a single-lane longitudinal safety evaluation that finds that the speed is unreasonable, the vehicle’s speed would be unreasonable.

### 2.3. LSTM

Compared to other algorithms, the prediction and classification of long short-term memory (LSTM) has high accuracy [25,26,27]. The LSTM neural network training model, which is a deep neural network with multiple hidden layers, has a memory function.

The LSTM memory function is provided by LSTM blocks in the network. The input data of the LSTM block are composed of the unit state $c_{t-1}$, the output $h_{t-1}$, and the input $x_{t}$ of the time delivered from time t−1, and the output data are the unit state $\;c_{t}$ and output $h_{t}$. At time t, LSTM has three doors: the forgetting gate $f_{t}$, the input gate $i_{t}$, forgetting gate and output gate $o_{t}$, respectively, where the forgetting gate determines the degree of state unit $c_{t-1}$ influence at t−1, the input gate determines how much input $x_{t}$ is reserved at time t, the output gate determines how much the state unit $\;c_{t}$ at time t keeps entering the output $h_{t}$, and $c_{t}$ and $h_{t}$ participate in the LSTM calculation at t + 1 again. The specific calculation process inside LSTM is as follows.

The calculation process of the forgetting gate is shown in Equation (8):

(8) $$f_{t}=\mathrm{sigmoid}\left(Wf\cdot \left[h_{t-1},x_{t}\right]+b_{f}\right)=\mathrm{sigmoid}\left(W_{fh}\cdot h_{t-1}+W_{fx}\cdot x_{t}+b_{f}\right)$$

The calculation process of the input gate is denoted in Equation (9):

(9) $$i_{t}=\mathrm{sigmoid}\left(Wt\cdot \left[h_{t-1},x_{t}\right]+b_{i}\right)=\mathrm{sigmoid}\left(W_{ih}\cdot h_{t-1}+W_{ix}\cdot x_{t}+b_{i}\right)$$

Then, the current input unit status is supposed to be an input, as shown in (10):

(10) $${\tilde{c}}_{t}=\tanh(W_{c}\cdot \left[h_{t-1},x_{t}\right]+b_{c})=\tanh(W_{ch}\cdot h_{t-1}+W_{cx}\cdot x_{t}+b_{c})$$

when calculating the state unit, “∘” stands for the dot multiplication sign; this process is illustrated in Equation (11):

(11) $$c_{t}=ft\circ ct-1+it\circ \tilde{c}t$$

The information output is obtained through the output gate, and the process of calculating the output gate is shown in Equation (12):

(12) $$o_{t}=\mathrm{sigmoid}\left(W_{o}\cdot \left[h_{t-1},x_{t}\right]+b_{o}\right)=\mathrm{sigmoid}\left(W_{oh}\cdot h_{t-1}+W_{ox}\cdot x_{t}+b_{o}\right)$$

Finally, the final output of the LSTM is shown in Equation (13):

(13) $$h_{t}=o_{t}\circ \tanh(c_{t})$$

Sigmoid and tanh are two activation functions in the following form,

(14) $$\mathrm{sigmoid}\left(x\right)=\frac{1}{1+e^{-x}}$$

(15) $$\tanh(x)=\frac{e^{x}-e^{-x}}{e^{x}+e^{-x}}$$

## 3. Experiment

### 3.1. Experimental Site

**Figure 4:**
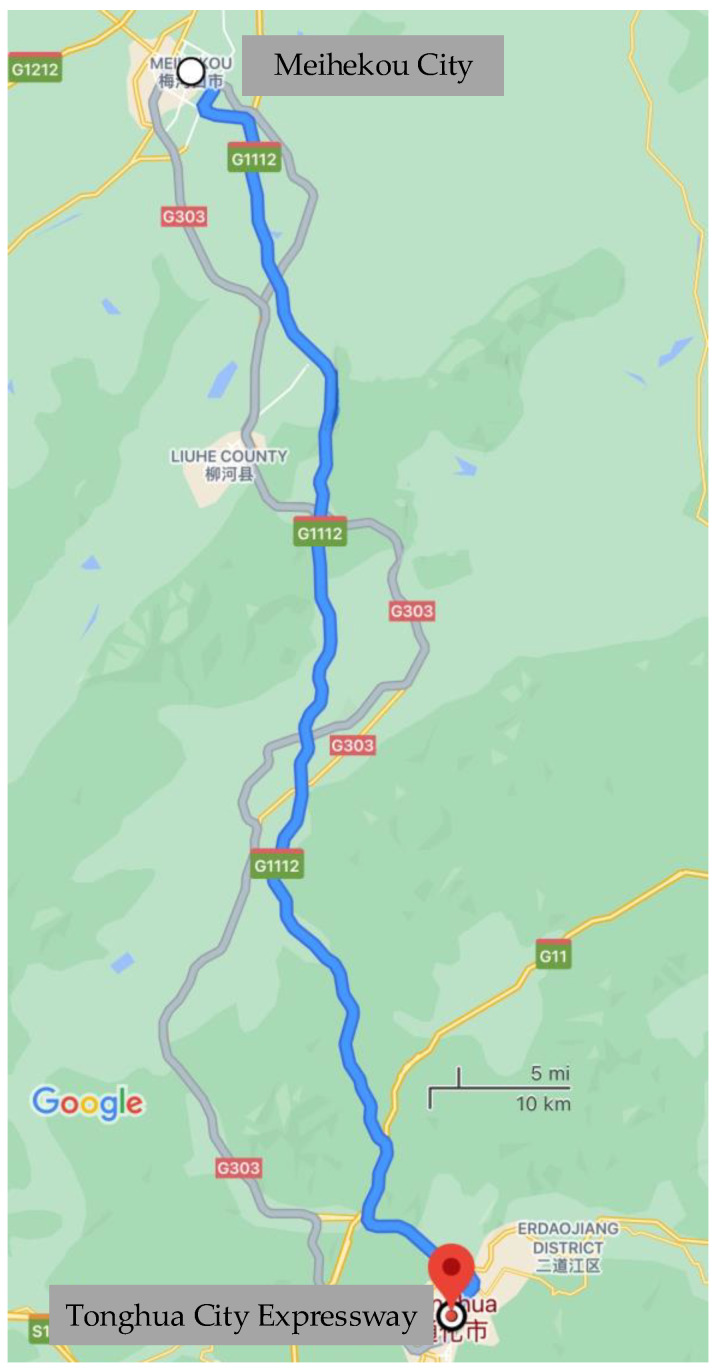
Experimental Roadmap.

The route used was from Meihekou City to Tonghua City Expressway, passing the Ji Shuang Expressway (G1112), in the Tonghua-Meihekou section of the Ji Shuang Expressway. The roadmap is shown in Figure 4. The test was conducted during off-peak hours to minimize traffic congestion problems. All tests were conducted under clear weather conditions to avoid adverse weather effects.

In this study, a road test of a real vehicle at four speeds was designed. The host vehicle was driven in the middle lane of a three-lane road. The host vehicle was driven at constant speeds of 60 km/h, 80 km/h, 100 km/h, and 120 km/h in highway traffic. The test vehicle was driven at each speed constantly for one hour. The tests started at 9 a.m., a non-peak traffic hour. During the test period, the road conditions and driving view was good, and all four sections of the experiment were driven along the designated route for real driving.

### 3.2. Experimental Equipment

**Figure 5:**
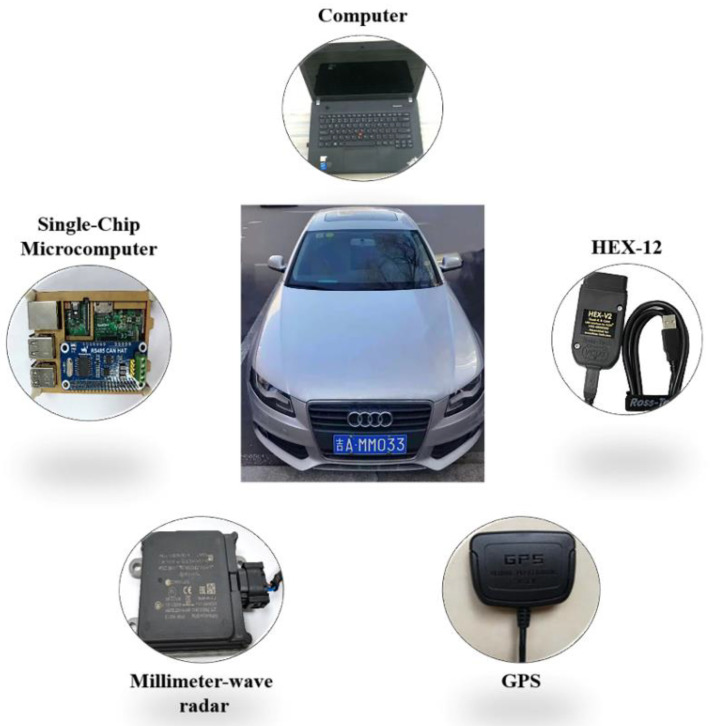
Experimental equipment.

The test equipment is shown in Figure 5. The test vehicle was an Audi A4L 2012 in good condition with no abnormalities in key systems (such as the engine, braking system, steering system, and driving system). A CAN bus analyzer, model HEX-V2, was used to record vehicle operating parameters in real time at a frequency of 1.67 Hz. There was a two millimeter-wave radar installed in the front and rear of the vehicle to measure the relative distance and speed between the main vehicle and the target vehicle. The model of two millimeter-wave radar is Continental ARS408. The vehicle was also equipped with a logger, which was used for the post-test classification of scenarios with or without a vehicle in front. All of these devices were connected to the same computer, and the start and end times of data acquisition were fully synchronized.

**Figure 6:**
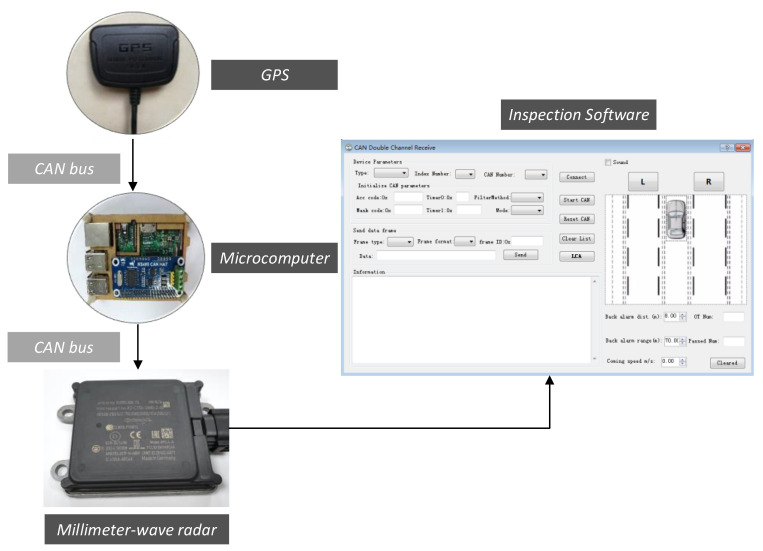
Diagram of millimeter-wave radar and GPS fusion.

We also integrated GPS with the millimeter-wave radar. The microcontroller (MCU) integrated with the CAN controller was selected to communicate with the CAN bus network through the terminals of the CAN bus transceiver in the MCU. A GPS speed meter was connected through the USB port of the MCU to analyze the data and obtain the speed of the moving vehicle. The data from the GPS was then integrated into the CAN bus of the millimeter-wave radar and then transmitted to the PC. The diagram of the fusion of millimeter-wave radar and GPS is shown in Figure 6.

### 3.3. Data Collection

The information regarding the collected longitudinal evaluation parameters is shown in Table 1. At the same time, the computer software developed in this study and described in Section 2.1.2 was used to detect whether a vehicle was overtaking or being overtaken in both lanes and to record the number of times that this occurred.

## 4. Discussion

### 4.1. Results of OTF

**Figure 7:**
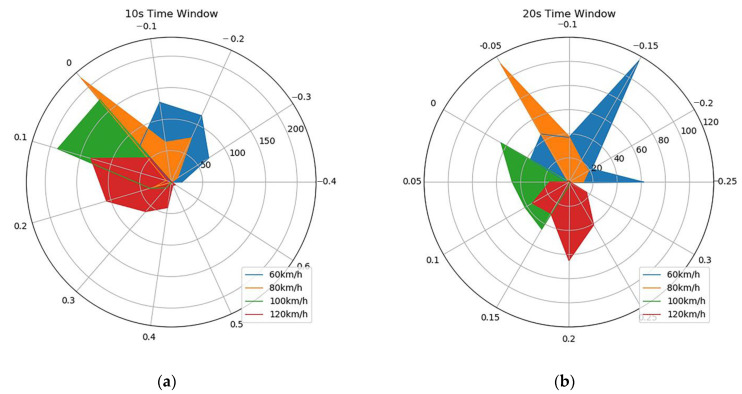

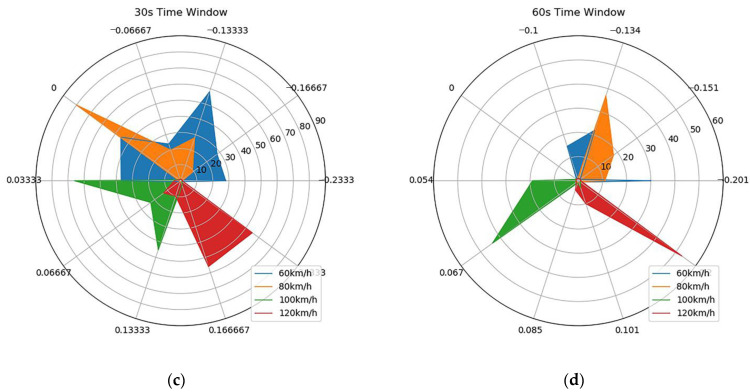
Radar chart of OTF data in different time windows at four vehicle speeds: (**a**) 10 s, (**b**) 20 s, (**c**) 30 s, and (**d**) 60 s.

After preprocessing raw data using linear transformation, the window width (δ = 10, 20, 30 and 60 s) was used to measure OTF data. Radar maps of 60, 80, 100, and 120 km/h OTF values for the four time windows are shown in Figure 7.

The ranges of detailed OTF values at different speed conditions in each time window are shown in Table 2.

At 60 and 80 km/h, the OTF range values are less than 0, as shown in Table 2. This means that the speed of the vehicle is much less than those in the other two lanes, and it is not safe to drive at a speed that is too slow in highway scenarios.

At a speed of 100 km/h, the OTF value fluctuates around 0, as seen in Figure 7a–d and from the OTF data in the table. From the definition of OTF, it can be seen that the state of the host vehicle is more in line with the current three-lane traffic flow. At the same time, the results also verify the validity of the OTF.

As shown in Figure 7a, when the time window is 10 s, the OTF at 120 km/h is more scattered, from 0 to 0.4, while under normal circumstances, a larger speed will cause the host car to be much faster than the vehicles’ speed in the other two lanes. Therefore, it is impossible to accurately judge the difference between the host car and the other two lanes at a higher speed when the time window of 10 s is selected. As shown in Figure 7d, at a speed of 120 km/h, the OTF values are more concentrated relative to the hourly values of the time window. Therefore, as the speed increases, the time window should be larger to ensure that the OTF accurately reflects the magnitude of the difference between the host car and the other two lanes.

As shown in Figure 7 and Table 2, when the width of the time window is larger, the OTF values corresponding to different vehicle speeds are more concentrated. Therefore, in the highway scenario, the width of the time window should be larger when making a selection.

On the basis of measured OTF values, the threshold [−a, a], which is proposed in Section 2.2, is calculated as (−0.085, 0.02):

(16) $$-a=\frac{\sum (X_{40}+X_{20})}{2n}\approx -0.085$$

(17) $$a=\frac{\sum (X_{40}+X_{60})}{2n}\approx 0.02$$

### 4.2. Results of the Model

In order to test the accuracy and recognition efficiency of the speed selection algorithm designed in this paper, the proposed LSTM-based speed evaluation algorithm was implemented in MATLAB using the real vehicle operating parameters collected in the real vehicle test to check its comprehensive performance.

A driver participated in real-vehicle experiments and made decisions regarding the driving conditions based on their personal driving experience. In this experiment, a total of 3594 valid samples were collected, of which 1719 groups were used as the training set, and the remaining 1875 groups were used as the test set for model training.

#### 4.2.1. Case 1: No Traffic in Front of the Vehicle

**Figure 8:**
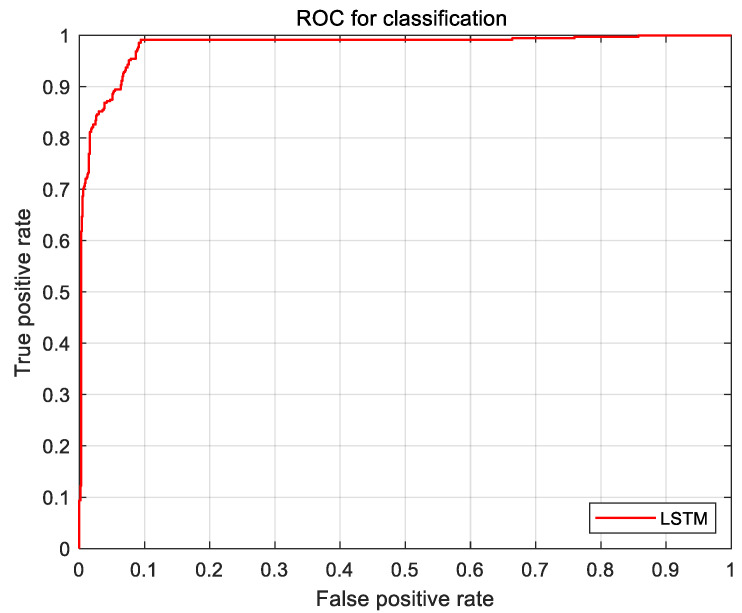
Receiver operating characteristic (ROC) curves for the case of no traffic in front of the vehicle.

According to the logbook, the experiment was divided into scenarios with and without a leading car. The 2245 valid samples without cars in front were divided into two data sets of suitable and unsuitable according to the OTF-based speed suitability evaluation model established in Section 2.2.1. The LSTM algorithm was predominantly used for model training; the result is shown in Figure 8.

#### 4.2.2. Case 2: Current Lane with Car in Front

**Figure 9:**
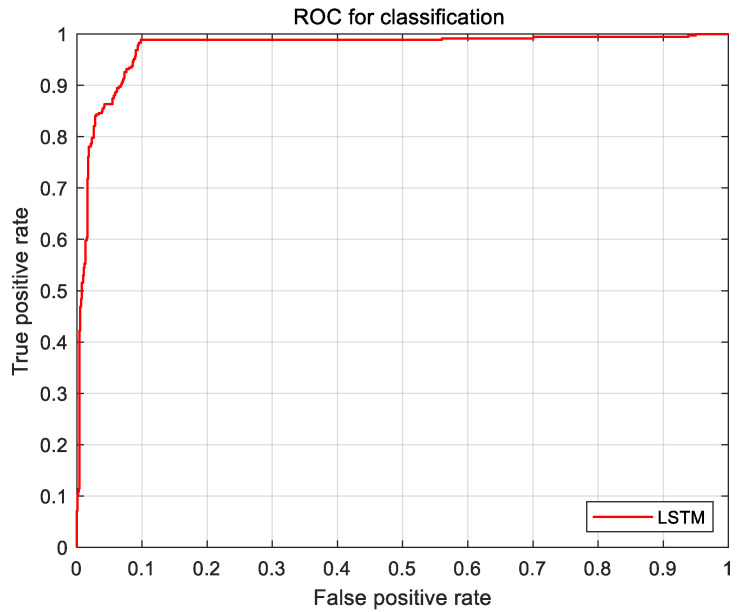
ROC curves for the case of the current lane having a car in front of the vehicle.

A valid sample of 1449 scenarios was collected with the presence of a car in front in the vehicle’s lane. The suitable and unsuitable data sets are divided according to the OTF-based vehicle speed suitability evaluation method described in Section 2.2.2. Then, the divided data were used as input labels. The training results of the LSTM model are shown in Figure 9.

The support vector machine (SVM) algorithm and Bayesian classification algorithm were used for comparison, as shown in Table 3.

Figure 8 below shows the receiver operating characteristic (ROC) curves for the three evaluation models, where these curves deviate from the 45° slope. The results show that, of the three models, the LSTM model has the highest accuracy.

**Figure 10:**
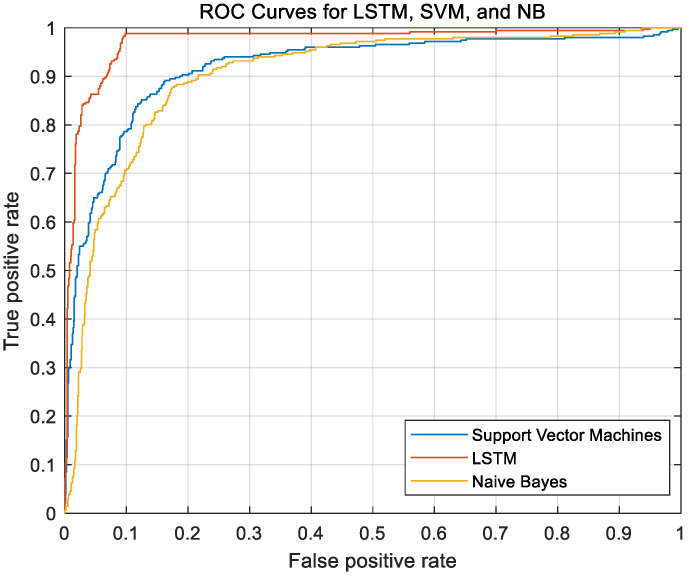
ROC curves for three models.

After the verification of the LSTM model test, the accuracy was 93%, and the effectiveness of the system was finally determined. The introduction of OTF as an observation variable ensured both the accuracy and efficiency of identification. The validation results are shown in Figure 10.

## 5. Conclusions

In this paper we studied the judgment of driving speed in a mixed traffic flow formed by autonomous vehicles and ordinary vehicles and proposed the new concept of overtaking frequency (OTF). Adopting a hierarchical evaluation method, an algorithm based on OTF parameters was developed at horizontal level. The algorithm was used to evaluate the suitability of a vehicle’s speed. At the same time, the algorithm was combined with longitudinal safety evaluation parameters for further judgment. The efficacy of the algorithm was also evaluated with scenarios based on the presence or absence of a leading vehicle in the main vehicle’s lane. The evaluation methods in the two scenarios of whether the front car exists in the current lane of the host car are different. Finally, the LSTM model was used to verify the accuracy and validity of the algorithm’s performance through road tests, and the following conclusions were reached.

Based on the comparison between the traffic flow of the left and right two lanes and the host vehicle, the OTF parameter was designed. By fusing millimeter-wave radar and GPS, an algorithm was developed to detect the number of vehicles passing and passed on both sides of the vehicle. Software was developed to embed the algorithm. With OTF as the horizontal observation variable and the longitudinal driving parameters of the vehicle (THW, TTC, etc.) as the vertical observation variables, the LSTM model, which reflects the suitability of the vehicle speed, was built and trained to assist the vehicle to obtain the difference between its own driving state and the driving state of surrounding vehicles. In this paper, two algorithms, SVM and Bayesian, were used for comparison with the LSTM model. The accuracy of LSTM was 93%, the accuracy of SVM was 87%, and the accuracy of Bayesian classification was 83%. This method showed good intuition and effectiveness when judging the driving state of vehicles on the road. The research results introduce a new basis for an algorithm for use by vehicles to determine their driving status in the context of autonomous driving technology. In the future, this approach can provide a theoretical basis for further research on uncertain decision-making. In the next step of research, the hardware and algorithms developed in this paper will be applied to the network environment in the future, and the data collected by the hardware in this paper will be uploaded to the server through the network to improve the safety and efficiency of all traffic.

## Figures and Tables

**Table 1:** Input parameters of the model.

Symbol	Parameter	Unit	Meaning	Recording Mode
t	Time	s	Driving time	CAN bus
v	Velocity	km/h	The velocity of host vehicle	CAN bus
$a_{x}$	Longitudinal acceleration	$m/s^{2}$	The longitudinal acceleration of the host vehicle	CAN bus
*D*	Distance	m	The distance between the host vehicle and the leading vehicle	Millimeter-wave radar
*v_rel_*	Relative velocity	km/h	The relative velocity of the host vehicle and the leading vehicle	Millimeter-wave radar

**Table 2:** The range of OTF under 4 speed conditions with different time window-width.

Time Window-Width	OTF Range of Values			
	60 km/h	80 km/h	100 km/h	120 km/h
10 s	[−0.377,−0.100]	[−0.222, 0.100]	[0.000, 0.202]	[0.000, 0.401]
20 s	[−0.350,−0.152]	[−0.155, 0.100]	[0.000, 0.151]	[0.050, 0.322]
40 s	[−0.333,−0.202]	[−0.133, 0.033]	[0.033, 0.100]	[0.067, 0.233]
60 s	[−0.333,−0.202]	[−0.134, 0.000]	[0.054, 0.101]	[0.075, 0.202]

**Table 3:** Model recognition accuracy. SVM: support vector machine.

Total Number	LSTM Model	Bayes Model	SVM Model
3594	93%	83%	87%

## Data Availability

The data presented in this study are available on request from the corresponding author.
